# Single-molecule visualization reveals the damage search mechanism for the human NER protein XPC-RAD23B

**DOI:** 10.1093/nar/gkz629

**Published:** 2019-08-02

**Authors:** Na Young Cheon, Hyun-Suk Kim, Jung-Eun Yeo, Orlando D Schärer, Ja Yil Lee

**Affiliations:** 1 School of Life Sciences, Ulsan National Institute of Science and Technology, Ulsan 44919, Republic of Korea; 2 Center for Genomic Integrity, Institute for Basic Science, Ulsan 44919, Republic of Korea

## Abstract

DNA repair is critical for maintaining genomic integrity. Finding DNA lesions initiates the entire repair process. In human nucleotide excision repair (NER), XPC-RAD23B recognizes DNA lesions and recruits downstream factors. Although previous studies revealed the molecular features of damage identification by the yeast orthologs Rad4-Rad23, the dynamic mechanisms by which human XPC-RAD23B recognizes DNA defects have remained elusive. Here, we directly visualized the motion of XPC-RAD23B on undamaged and lesion-containing DNA using high-throughput single-molecule imaging. We observed three types of one-dimensional motion of XPC-RAD23B along DNA: diffusive, immobile and constrained. We found that consecutive AT-tracks led to increase in proteins with constrained motion. The diffusion coefficient dramatically increased according to ionic strength, suggesting that XPC-RAD23B diffuses along DNA via hopping, allowing XPC-RAD23B to bypass protein obstacles during the search for DNA damage. We also examined how XPC-RAD23B identifies cyclobutane pyrimidine dimers (CPDs) during diffusion. XPC-RAD23B makes futile attempts to bind to CPDs, consistent with low CPD recognition efficiency. Moreover, XPC-RAD23B binds CPDs in biphasic states, stable for lesion recognition and transient for lesion interrogation. Taken together, our results provide new insight into how XPC-RAD23B searches for DNA lesions in billions of base pairs in human genome.

## INTRODUCTION

Nucleotide excision repair (NER) is a highly conserved DNA repair pathway in charge of eliminating a diverse repertoire of DNA damage such as ultraviolet (UV) light-induced photo-lesions, intrastrand crosslinks and bulky adducts derived from various carcinogens (1,2). In mammals, about thirty different proteins involved in NER remove DNA lesions in an orchestrated manner (1). Defects in human NER cause hereditary diseases such as xeroderma pigmentosum, which is characterized by UV sensitivity and an extreme predisposition to skin cancer (3). NER operates in two sub-pathways, transcription-coupled NER (TC-NER) and global genome NER (GG-NER). In TC-NER, an RNA polymerase stalled at a lesion during transcription serves as a DNA damage indicator. In GG-NER, xeroderma pigmentosum complementation group C protein (XPC) along with RAD23B and Centrin2 recognizes a variety of NER substrates by sensing the local distortion and/or thermodynamic destabilization of the DNA helix caused by the modified bases (4,5). After finding a lesion, XPC recruits TFIIH, which verifies the chemical modification of the NER substrate and opens a bubble around the lesion site using the activity of its two helicase subunits XPB and XPD. Subsequently, XPA and RPA join the complex, stabilize the open DNA bubble, and fully assemble the NER machinery (6,7). XPF-ERCC1 and XPG make incisions on the 5′ and 3′ sides of the lesion on the damaged strand, respectively, resulting in removal of the lesion (8). The NER process is completed by filling in the gap by DNA polymerases and sealing the nick by DNA ligases (1).

The search for damage by XPC is essential and critical for NER because it initiates the entire process and is a rate limiting step (9). Insights into the molecular mechanism of damage recognition by XPC were gained from structural studies of the yeast XPC ortholog Rad4 (10–12). The crystal structure of Rad4 bound to a cyclobutane pyrimidine dimer (CPD) in a mismatch or a 6–4 photoproduct (6–4 PP) revealed that Rad4 captures the undamaged strand at the lesion site while the lesion is flipped out of the helix and does not directly interact with Rad4 (10,12). Binding the undamaged strand accounts for the ability of XPC to detect a wide spectrum of structurally diverse lesions. Surprisingly, the crystal structure of Rad4 immobilized on undamaged duplex DNA through a linker showed a very similar binding mode to that on CPD, suggesting that Rad4 searches for damaged DNA by kinetic gating mechanism (11). Recently, single-molecule tight-rope assay was used to address the question of how Rad4 locates DNA lesions (13). Kong *et al.* found that Rad4 searches for NER substrates within a long DNA via one-dimensional (1D) diffusion with three types of motion of Rad4: random motion, constrained motion and immobile state, on UV-damaged DNA. They attributed the random motion and the immobile state to 1D free diffusion and stable lesion-binding state, respectively. For the constrained motion restricted within a couple of thousands base pairs, they proposed that CPDs as poor NER substrates cause a conformational change of Rad4, which thereby increases the diffusion energy barrier between DNA and Rad4, rendering the motion of Rad4 more restricted around CPDs. They suggested that the constrained motion can mark CPD sites as a first response and termed this process ‘recognition-at-a-distance.’ However, the molecular basis for constrained motion was not elucidated and it is unclear how it would be compatible with the proposed kinetic gating mechanism.

Several mechanisms for target search of DNA binding proteins, including DNA damage, have been suggested (14–18). Among them, a diffusion-driven search mechanism called ‘facilitated diffusion’ is thought to speed up the search, especially at low protein concentrations. For 1D diffusion along DNA, there are two sub-pathways, ‘sliding’ and ‘hopping’ (19). Sliding is the 1D motion along DNA contour while maintaining continuous contact with DNA. By contrast, hopping is the three-dimensional motion of protein through repeated microscopic dissociation and re-association with the DNA. An advantage of the sliding mode is that it allows proteins to survey DNA sequences. Hence, transcription factors such as lac repressor and some repair proteins slide along DNA to recognize their target bases (20–22). In contrast, the hopping mechanism allows a protein to pass through protein obstacles on DNA (17,23–24). These two mechanisms can be experimentally distinguished (19). In the hopping mode, a protein needs to be physically detached from DNA, and cations can be re-condensed on the DNA phosphate backbone. Therefore, the speed of diffusion via hopping increases at higher salt concentrations whereas the diffusion via sliding is insensitive to ionic strength.

In this paper, we examined the damage search mechanism of human XPC-RAD23B using DNA curtain, a high-throughput single-molecule imaging technique. Like Rad4-Rad23, XPC-RAD23B displayed the three types of motions on either damaged or on undamaged DNA- diffusive, constrained and immobile. We found that the location of constrained motion is highly correlated with consecutive AT-tracks, suggesting that the constrained motion is induced by local DNA instability and can occur independently of DNA lesions. The increase of diffusion coefficients with ionic strength suggested that XPC-RAD23B diffuses on DNA via hopping rather than sliding. Our collision experiments demonstrated that the hopping facilitates lesion search by bypassing protein obstacles on DNA. At last, we examined how XPC-RAD23B identifies CPDs. Our results showed that XPC-RAD23B recognizes CPDs with low efficiency and can exhibit transient and stable binding to CPDs.

## MATERIALS AND METHODS

All the details about our experiments are described in the Supplementary Data. Here we briefly describe the materials and methods

### DNA preparation

All oligomers except CPD-containing one were synthesized from Bioneer (South Korea) (Supplementary Table S1). Lambda phage (λ) DNA for the undamaged DNA substrate was purchased from New England Biolabs. For the lesion-containing λ-DNA, CPD-containing oligomer (λ-I3_CPD) was synthesized from Gene Link (USA) (Supplementary Table S1). To insert the lesion in the λ-DNA, the specially engineered λ-I3 was used, which contains seven nickase sites at a specific region (16) (Supplementary Figure S1A). Nickase treatment and subsequent heating at 65°C generated a gap consisting of three repeated sequences on the λ-I3. Excessive CPD-containing oligomers were annealed to the gap and sealed by ligation to produce λ-DNA containing three repeats of CPDs. The CPD insertion was confirmed by NcoI digestion (Supplementary Figure S1B and C). For the DNA curtain assay, all λ-DNA molecules were ligated with Lambda biotin-L and Lambda dig-R (or Lambda biotin-R and Lambda dig-L) (Supplementary Table S1).

### Protein preparation

Full length of XPC-RAD23B with 3×FLAG at N-terminus was over-expressed in Sf9 insect cells. After cell harvest and lysis, XPC-RAD23B was purified through anti-FLAG M2 affinity agarose beads (A2220, Sigma), gel filtration (HiLoad16/600 Superdex 200, Pharmacia) and heparin columns (HiTrap Heparin, Pharmacia) (Supplementary Data).

As a protein roadblock for the collision with XPC-RAD23B, catalytically inactive EcoRI mutant (EcoRI^E111Q^), which has 3×FLAG peptides, was purified as described previously (25). The detail for this purification is also described in Supplementary Data.

### DNA curtain assay

**Figure 1.**
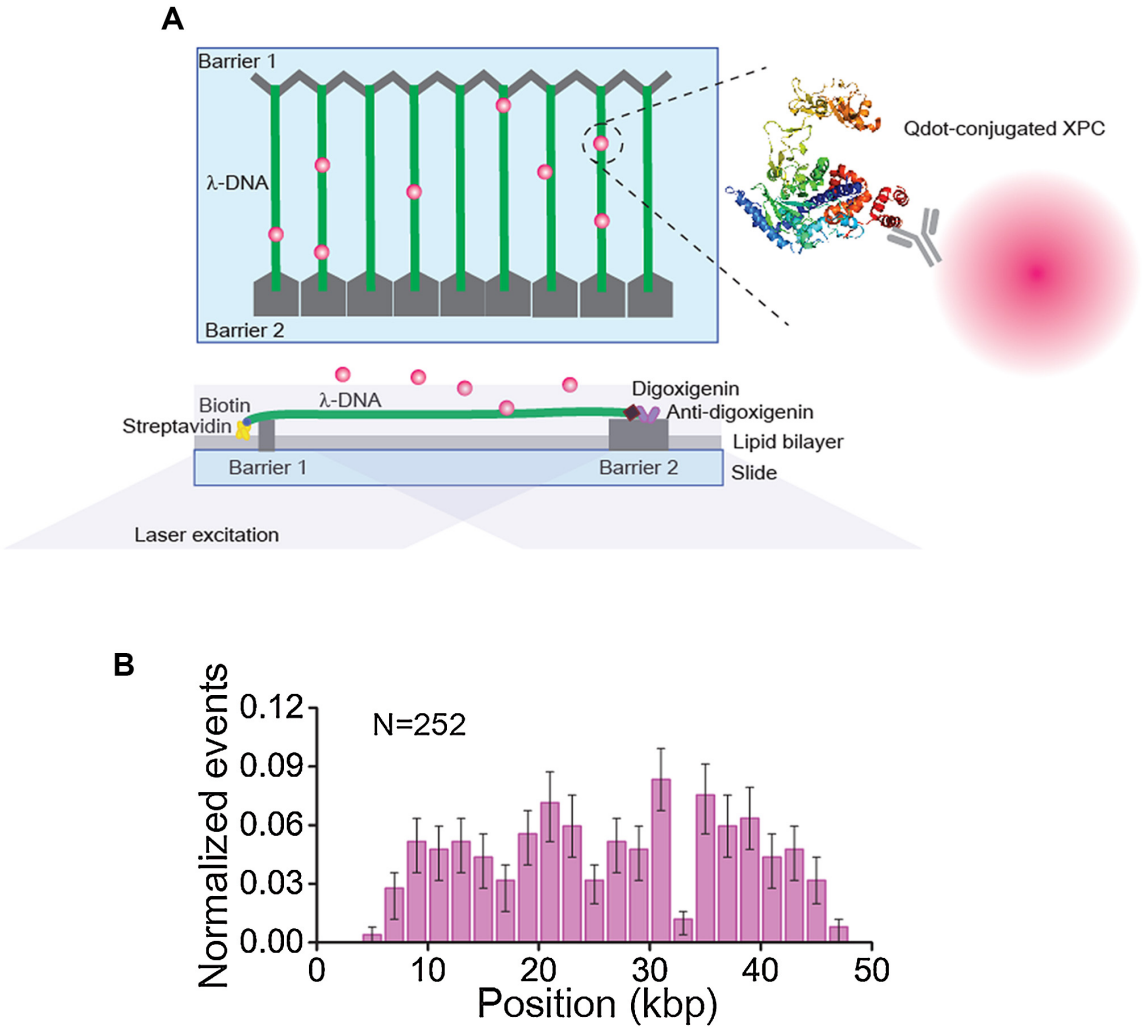
Schematic of DNA curtain assay with quantum dot (Qdot)-conjugated XPC and initial binding position on undamaged lambda (λ) DNA. (**A**) Schematic of DNA curtains. Top left: top view of DNA curtains, bottom left: side-view of DNA curtains, and right: Qdot-conjugated XPC. The structure of XPC is adopted from yeast Rad4-Rad23 (10). (**B**) Histogram for the initial binding positions of XPC-RAD23B on undamaged λ-DNA. The error bars were obtained by bootstrapping with 70% confidence interval.

For the DNA curtain assay, the total internal reflection fluorescence microscope combined with a fluidic system was built-up as described previously (25). A nano-patterned fused silica slide was assembled into a flowcell containing microchamber. Liposomes consisting of DOPC (1,2-dioleoyl-*sn*-glycero-3-phosphocholine), 0.5% biotinylated-DOPE (1,2-dioleoyl-*sn*-glycero-3-phosphoethanolamine-N-(cap biotinyl)) and 8% mPEG 2000-DOPE (1,2-dioleoyl-*sn*-glycero-3-phosphoethanolamine-N-[methoxy(polyethylene glycol)-2000]) (Avanti Polar Lipids) were deposited to the surface of flowcell to form a lipid bilayer (25). A total of 0.1 mg/ml of anti-digoxigenin (11214667001, Roche) was injected and adsorbed on the pentagonal nano-structure. After further surface passivation by 0.4% of bovine serum albumin, 1 mg/ml streptavidin was introduced. Then either undamaged or CPD-containing λ-DNA molecules tagged with biotin and digoxigenin at opposite ends were anchored on individual lipid molecules via biotin-streptavidin linkage. Under the continuous flow, the digoxigenin at the other end was tethered to the pentagonal nano-barrier. Thereby the DNA molecules remained stretched when the flow was turned off (Figure 1A).

All XPC-RAD23B experiments on the DNA curtain were carried out in the XPC buffer (25 mM Tris–HCl [7.5] with 40 mM, 100 mM or 150 mM NaCl) at 23°C. XPC-RAD23B was fluorescently labeled by FLAG-antibody-conjugated quantum dot (Qdot), which was prepared from a commercial kit (S10454, Thermo Fisher Scientific). XPC-RAD23B and FLAG-antibody-Qdots were mixed at 1 to 40 molar ratio and then incubated on ice for at least 15 min. Excessive Qdots ensured that only one XPC-RAD23B is conjugated with a single Qdot. About 0.5–1 nM XPC-RAD23B was injected into the flowcell. For the collision experiments, 3×FLAG-tagged EcoRI^E111Q^ and FLAG-antibody-Qdot having different emission wavelength (605 nm) were conjugated at 1–10 molar ratio on ice for at least 15 min. Then 3 nM EcoRI^E111Q^ was injected into the flowcell and incubated with DNA in EcoRI buffer (40 mM Tris–HCl [7.5], 50 mM NaCl and 2 mM MgCl_2_). After unbound EcoRI^E111Q^ was completely washed out, 1 nM XPC-RAD23B tagged with Qdot (705 nm emission) was injected in the XPC buffer containing 150 mM NaCl. When the maximum amount of proteins reached the DNA curtains, the flow was turned off and data were collected through NIS-elements (Nikon) with 100 ms exposure time for 5 min. Although all Qdots were conjugated with FLAG antibodies and both proteins had FLAG tags, we did not observe any adhesion of two Qdots on DNA, ensuring that there was no serious cross binding between proteins and Qdots.

For the binding test of XPC-RAD23B on the CPD-containing λ-DNA, single-tethered DNA curtain assay was performed, in which the biotinylated end of λ-DNA is anchored on lipid bilayer and the opposite end is free. To stretch the CPD-containing λ-DNA, XPC buffer with 150 mM NaCl was continuously flowed in. A total of 1 nM Qdot-conjugated XPC-RAD23B was injected. After 10 min incubation with the damaged λ-DNA in the absence of flow, the binding of XPC-RAD23B was imaged under the flow.

### Data analysis

All data were transformed into TIFF format and then analyzed by ImageJ software (NIH). The motion of single XPC-RAD23B was tracked by an ImageJ plug-in, MosaicSuite particle tracker. For the molecules that bound to DNA after collecting data, the initial binding positions were obtained by taking the position coordinates of the first frame for each particle tracking data. The locations of constrained motion were gained from the mean of the restricted fluctuation. Diffusion coefficients were estimated by linear regression of the first three data points of mean square displacement (Supplementary Data). On the other hand, the distribution of consecutive AT-tracks in λ-DNA was calculated by Matlab (Mathworks) with the λ-DNA sequence provided by Gene Bank.

### Electrophoretic mobility shift assay (EMSA)

39-mer CPD-containing dsDNA and homoduplex DNA were prepared, both of which were labeled with Cy3 (Supplementary Data andSupplementary Table S1). A total of 10 nM of each DNA was incubated with XPC-RAD23B at different concentrations in 25 mM Tris–HCl [7.5] and 150 mM NaCl at 23°C for 20 min. The reaction mixture was analyzed by electrophoresis in 5% non-denaturing polyacrylamide gel at 4°C. The gel was scanned by Typhoon RGB (GE Healthcare), and the band intensity was quantified by ImageJ (NIH).

## RESULTS

### XPC-RAD23B exhibits three distinct classes of motion on undamaged DNA

Full length XPC-RAD23B tagged with a 3×FLAG peptide at the N-terminus of XPC was purified and labeled with a FLAG-antibody-conjugated Qdot (Figure 1A and Supplementary Figure S2A). Our XPC-RAD23B was fully active, as demonstrated by its ability to complement the NER excision activity of a 1,3-cisplatin DNA adduct in an XPC-deficient cell extract (Supplementary Figure S2B). The protein also displayed robust DNA binding activity using electrophoretic mobility shift assay with oligonucleotide containing a CPD (Supplementary Figure S2C). Consistent with the previous studies, XPC-RAD23B bound to the CPD-containing DNA with a modest preference over undamaged DNA (26).

The behavior of XPC-RAD23B on DNA was visualized in real time using the single-molecule DNA curtain assay (Figure 1A). We mapped the initial binding positions of XPC-RAD23B on undamaged λ-DNA (27) and found that the binding distribution was random (Figure 1B), suggesting that the initial binding of XPC-RAD23B on DNA is sequence-independent.

**Figure 2.**
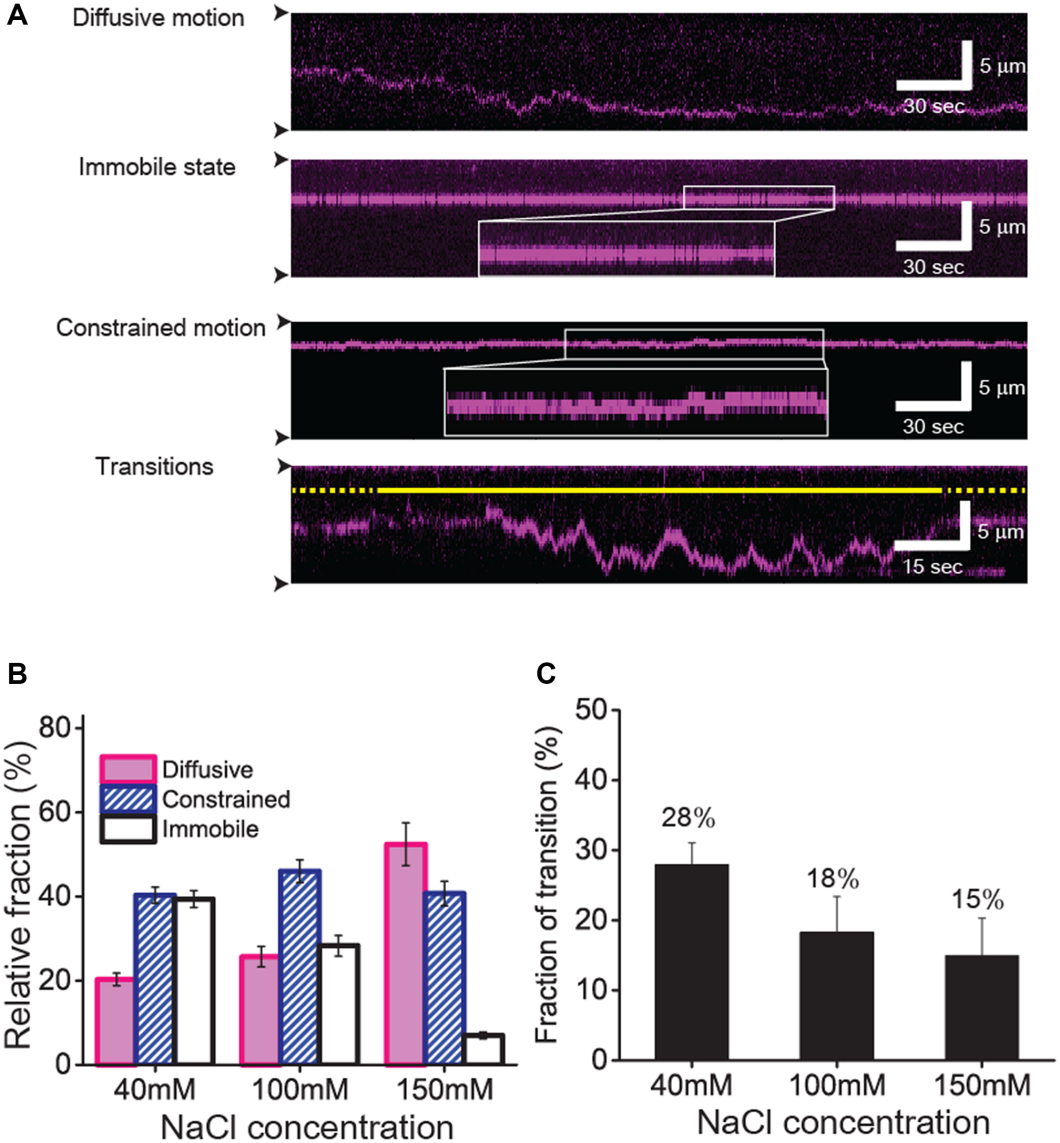
Three distinct types of motion of XPC-RAD23B on undamaged DNA (**A**) Kymographs for different types of motion of XPC-RAD23B. From top to bottom, diffusive motion, immobile state, constrained motion and transition between two distinct states are displayed. The white box in the kymographs showing the immobile state and constrained motion represents the zoom-in view of trace. The constrained motion is clearly distinguished from the immobile state. The solid and dotted yellow lines stand for diffusive motion and constrained motion, respectively. The black arrow heads indicate barrier positions. (**B**) Relative fraction of each motion according to salt concentration. The error bars were obtained by the standard deviation of multinomial distribution. (**C**) Relative fraction of transitions out of total traces according to salt concentration. The error bars were obtained by the standard deviation of binominal distribution.

Next, we examined the motion of XPC-RAD23B on undamaged DNA. Three distinct types of motion were observed at three different concentrations used (Figure 2A and Supplementary Figure S3). The first type of motion was random movement along DNA over long distances without any specific directionality (top of Figure 2A and Supplementary Movie S1). We assigned this movement as *diffusive* motion. The second type was the stable binding of XPC to one position on the DNA, in which XPC did not move on the kymograph. We designated this type of movement as *immobile* state (second of Figure 2A and Supplementary Movie S2). This immobile state was distinguished from the surface-stuck Qdot by lateral thermal fluctuation perpendicular to DNA stretch (Supplementary Figure S4). For the third type of movement, XPC moved on DNA, but the displacement of XPC was restricted to a short range of less than three pixels (±1.5 kbp) (third of Figure 2A and Supplementary Movie S3). We considered this limited movement as *constrained* motion. The reason that we set three pixels as a criterion for constrained motion was that this allowed us to clearly distinguish the motion from the immobile fraction. These three types of motion were consistent with the previous results obtained with Rad4 (13).

We then analyzed the relative population of each motion in dependence of the salt concentration (Figure 2B). The movement of XPC-RAD23B on DNA was highly dependent on the ionic strength. At 40 mM NaCl, the constrained motion and immobile state (∼40% each) were more populated than the diffusive motion (∼20%). Increasing the salt concentration led to a reduction of the immobile fraction with a concomitant increase of the diffusive fraction. At 150 mM NaCl, the immobile state was dramatically suppressed (∼7%), whereas there was an over twofold increase in the population of diffusive molecules (∼52%). This ionic strength dependence implies an electrostatic interaction between XPC-RAD23B and duplex DNA. It is possible that DNA lesions could lead to the immobilization of the protein. However it has been previously shown that the binding stability of XPC-RAD23B to lesions is not affected by ionic strength (28). If the immobile state were due to accidental DNA lesions on the λ-DNA, then the population of immobile species should be less sensitive at higher salt concentrations of 150 mM NaCl. Our data ascertain that the immobile state does not stem from inadvertent DNA damage.

Importantly, we observed transitions between different types of motion at all tested concentrations of NaCl (bottom of Figure 2A and Supplementary Figure S3, and Figure 2C). Most transitions occurred between diffusive motion and constrained one (>85%) regardless of salt concentrations, and the transition frequency modestly decreased with increasing ionic strength.

### The location of constrained and immobile states is highly correlated with consecutive AT-tracks

**Figure 3.**
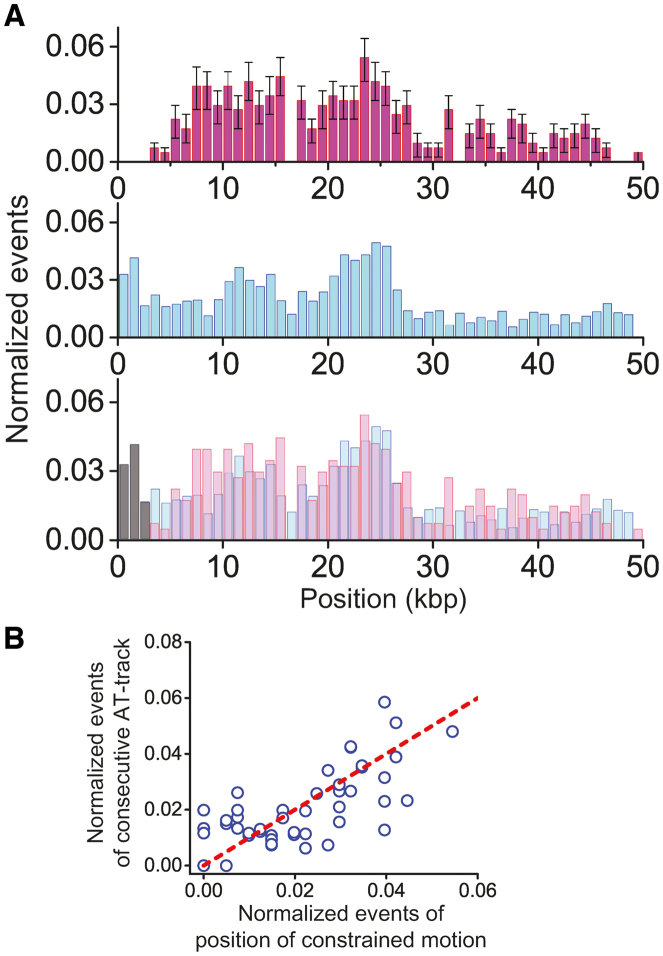
Nature of constrained motion (**A**) The position distribution of the constrained motion and immobile state and locations of consecutive AT-tracks in λ-DNA. Top: the distribution histogram of positions where both constrained and immobile species appear on undamaged λ-DNA. Middle: the distribution histogram of consecutive AT-tracks greater than 4 bp in the λ-DNA (bin size: 1 kbp). Bottom: the overlap of the above two histograms. (**B**) Correlation analysis of the positions of constrained and immobile species relative to the locations of consecutive AT-tracks. The red dashed line represents the perfect positive correlation. Pearson correlation coefficient is 0.7 with 10^−8^*P*-value.

As indicated above, the immobile state was neither due to stable binding to accidental DNA lesions nor to nonspecific surface-adsorption. Instead, we considered the possibility that the immobile species actually move on DNA within a distance of less than 1 kbp, which cannot be measured with our spatial resolution (∼1 kbp per pixel). To test this possibility, we compared the locations of immobile species and those of constrained motion on λ-DNA and found that they overlapped with a high correlation (correlation efficient: 0.6, *P*-value = 10^−7^), suggesting that the causes of constrained motion and immobile state are related (Supplementary Figure S5A and B). Interestingly, constrained motion was more prominent on the first half of λ-DNA (top of Figure 3A). When we reversed the orientation of the λ-DNA by switching the biotin and digoxigenin ends, the distribution was also reversed (Supplementary Figure S5C and D), confirming that the biased distribution is due to the intrinsic interaction between XPC-RAD23B and λ-DNA. Given the fact that first half of λ-DNA is AT-rich, we reasoned that AT-rich sequences might correlate with the constrained motion (29). We counted AT-tracks longer than 4 bp that are defined as consecutive sequences consisting of only As and Ts, because the crystal structure of Rad4 revealed the protein flips two nucleotides of undamaged strand opposite of the lesion and contacts with four bases around a DNA lesion (10). The distribution of AT-tracks was also biased in the half region of the λ-DNA (middle of Figure 3A). Remarkably, the positions of AT tracks were well-overlapped with locations of the constrained and immobile species (bottom of Figure 3A). The Pearson correlation coefficient of the two distributions was 0.7 (*P*-value = 10^−8^), suggesting that consecutive AT-tracks are highly correlated with the constrained and immobile species (Figure 3B). Taken together, our results suggest that the constrained motion and the immobile state are of the same nature and are enriched in sequences of decreased duplex stability.

### XPC-RAD23B diffuses on DNA via hopping

**Figure 4.**
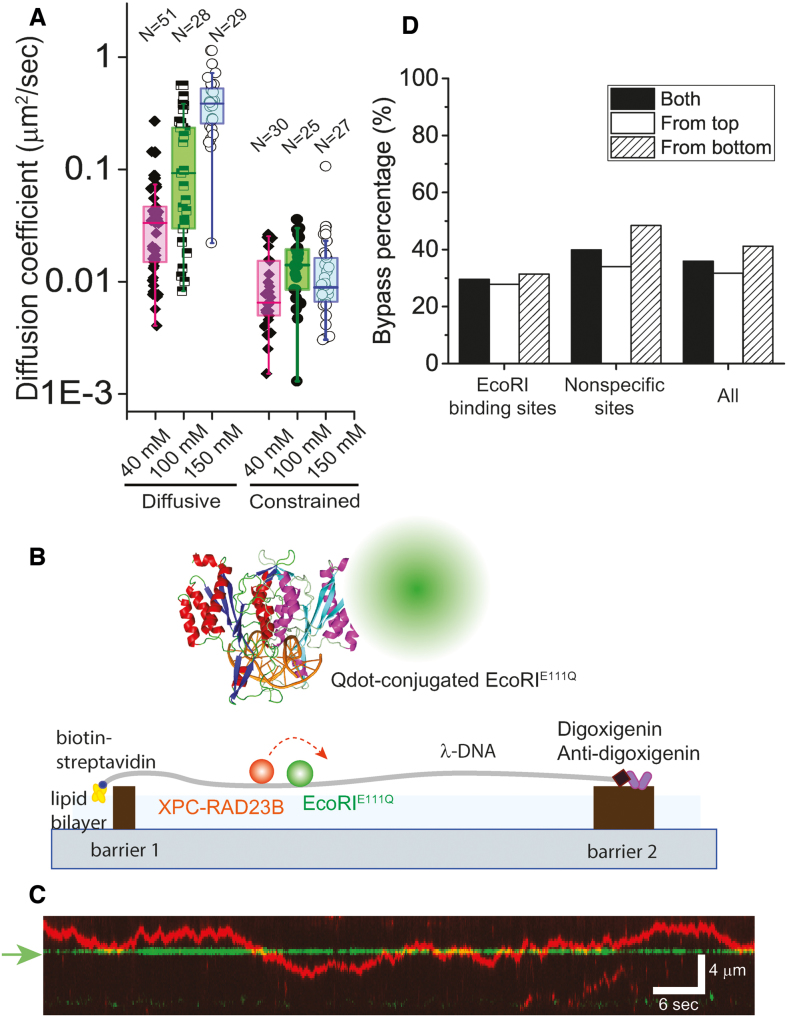
Diffusion coefficients of XPC-RAD23B according to salt concentrations and collision between XPC-RAD23B and roadblock protein EcoRI^E111Q^ at 150 mM NaCl. (**A**) Box plots of diffusion coefficients of diffusive motion (*D*_diff_) and constrained motion (*D*_cons_) at different NaCl concentrations (N: number of molecules). *D*_diff_ at 40, 100 and 150 mM NaCl was 0.034 ± 0.045 μm^2^/s (∼5.4 × 10^5^ bp^2^/s), 0.093 ± 0.15 μm^2^/s, (∼1.5 × 10^6^ bp^2^/s) and 0.39 ± 0.23 μm^2^/s (∼6.2 × 10^6^ bp^2^/s) (median ± SD), respectively. *D*_cons_ was 0.010 μm^2^/s at 40 mM NaCl, 0.014 ± 0.010 μm^2^/s at 100 mM NaCl and 0.010 ± 0.020 μm^2^/s at 150 mM NaCl. (**B**) Schematic of DNA curtain experiment for the collision between XPC-RAD23B and EcoRI^E111Q^. EcoRI structure is adopted from protein data bank (PDB ID: 1CL8). (**C**) Kymograph for the collision between XPC-RAD23B (red) and EcoRI^E111Q^ (green). The green arrow represents EcoRI cognate site on λ-DNA. (**D**) Quantitative analysis for the bypass events at the collision according to the collision orientation. The bypass percentage was estimated for EcoRI^E111Q^ bound at either cognate (*N* = 290) or non-specific sites (*N* = 423).

We then quantitatively analyzed the motion of XPC-RAD23B using the time trajectory for each molecule obtained from the single particle tracking (Supplementary Figure S6A, B, E and F). The relative displacements between adjacent frames fit well to a Gaussian function with its center around zero, indicating that it occurred by Brownian motion (Supplementary Figure S6C and G). The diffusion of XPC-RAD23B was quantitatively assessed by estimating the diffusion coefficient (*D*), which did not significantly vary with the number of fitted data points (Supplementary Figure S6D, H and I). The diffusion coefficient of diffusive motion (*D*_diff_) increased steeply with increasing ionic strength (Figure 4A). The *D*_diff_ at 150 mM was approximately 10 times higher than at 40 mM NaCl. This dramatic escalation of *D*_diff_ with increasing salt concentration suggests that XPC-RAD23B diffuses along DNA via *hopping*. In contrast to *D*_diff_, the diffusion coefficient of constrained motion (*D*_cons_) was rarely affected by ionic strength. Inferred from the high correlation between the constrained motion and AT tracks, an interaction between XPC-RAD23B and AT-tracks may result in slow diffusion independent of ionic strength.

### XPC-RAD23B can bypass protein obstacles on DNA

It has been known that 1D diffusion via hopping facilitates the bypass over protein roadblocks on DNA (17,23–24). Therefore, if XPC-RAD23B diffused via hopping, it should be able to bypass protein obstacles. To test this possibility, we asked whether XPC-RAD23B could bypass other proteins that were bound to the same DNA molecules (Figure 4B). We used catalytically inactive EcoRI mutant (EcoRI^E111Q^) as a protein roadblock, which binds tightly to but does not cleave its cognate site (30). EcoRI^E111Q^, labeled with Qdot (605 nm), was visible on the DNA curtains at defined positions (Figure 4C). When XPC-RAD23B encountered EcoRI^E111Q^ at physiological salt concentration (150 mM NaCl), it either reversed direction or bypassed the EcoRI^E111Q^ obstacle (Figure 4C and Supplementary Figure S7). We observed short periods of colocalization of the two proteins on DNA but did not observe any evidence that XPC-RAD23B could either push or evict EcoRI^E111Q^ from the DNA. The bypass probability was not significantly affected by the collision orientation consistent with the symmetrical binding of EcoRI^E111Q^ to DNA (Figure 4D). The overall bypass probability was 30% and 40% for the EcoRI^E111Q^ bound to cognate and nonspecific sites, respectively. Such a high bypass probability indicates that the XPC-RAD23B can frequently bypass DNA-bound protein obstacles.

### XPC-RAD23B recognizes CPDs with low efficiency

**Figure 5.**
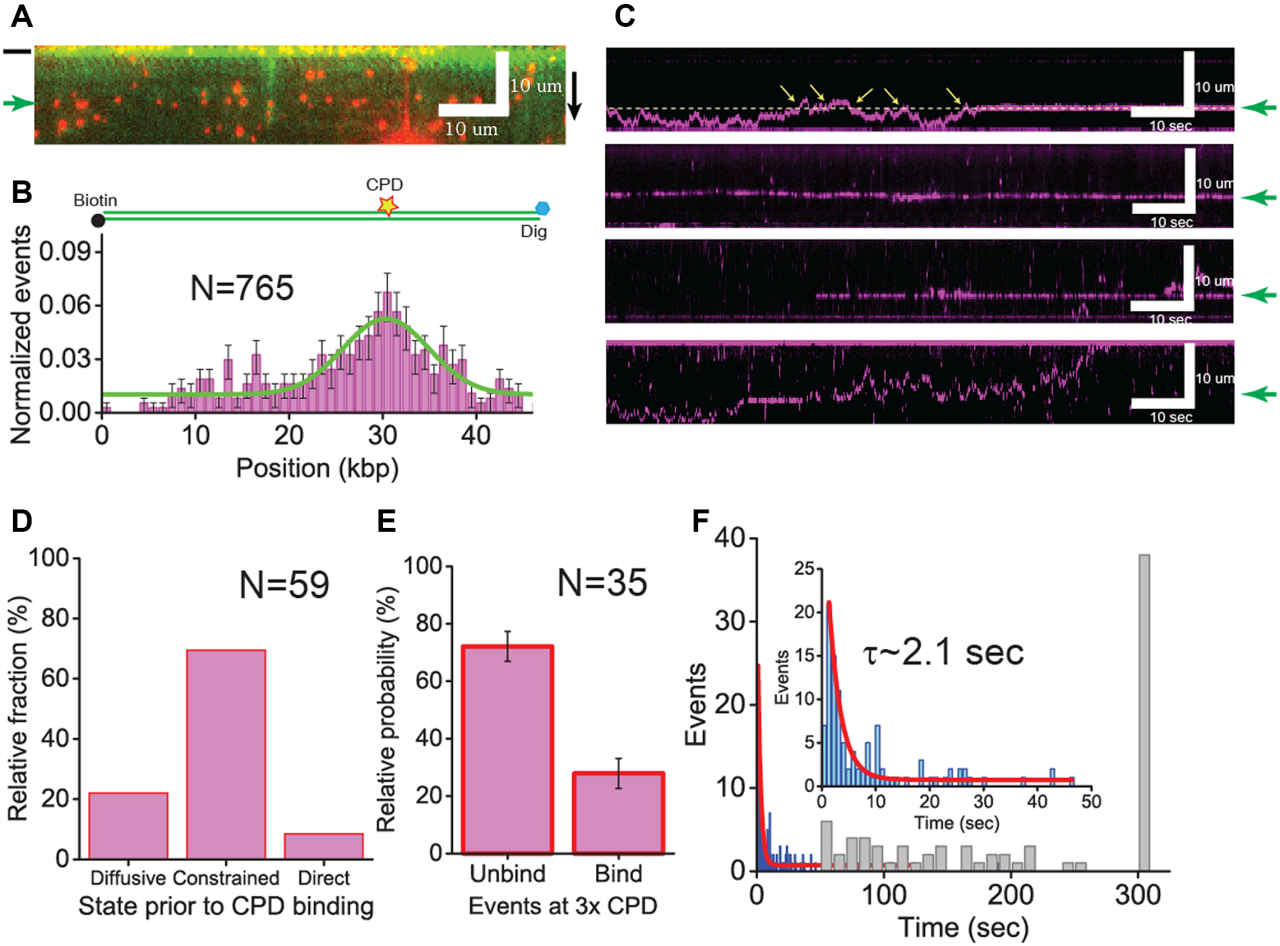
XPC-RAD23B on the damaged DNA (**A**) Snap shot of single-tethered DNA curtain for the specific binding of XPC-RAD23B on CPD-modified λ-DNA. XPC-RAD23B molecules (red) are aligned at CPD sites (green arrow) on YOYO-1 stained λ-DNA (green). The black bar next to the image indicates the barrier position. The black arrow represents the flow orientation. (**B**) Histogram for binding positions of XPC-RAD23B on CPD-inserted λ-DNA. The histogram was fitted by a single Gaussian function (solid green line). The peak center is placed at 30.5 ± 3.5 kbp, which is close to the actual CPD location (33.5 kbp). The error bars were obtained by bootstrapping with 70% confidence interval. The yellow star represents the location of CPDs on the λ-DNA. (**C**) Kymographs showing the search of XPC-RAD23B for CPDs on λ-DNA. Top: CPD recognition by diffusive motion; second: CPD recognition by constrained motion; third: direct binding to CPDs; and bottom: transient binding to CPDs during diffusive motion. The green arrows next to kymographs indicate the location of CPDs. In the top panel, the yellow dashed line is an abstract line for the location of CPDs and yellow arrows represent the events that XPC-RAD23B does not identify CPDs. (**D**) Relative fraction of diffusive, constrained and direct binding to CPDs by XPC-RAD23B −22.0, 69.5, 8.5%, respectively. The total number of events (N) analyzed for relative fraction was 59. (**E**) The probability that XPC-RAD23B binds or misses CPDs when encountering the lesions. The binding probability represents CPD recognition efficiency of XPC-RAD23B in diffusive motion at CPDs. The error bar represents standard error. (**F**) Duration of binding of XPC-RAD23B to CPDs. Lifetimes were collected (blue histogram) and analyzed by fitting with a single exponential decay function (red line). The total number of molecules (N) analyzed for the lifetime was 135. The lifetime (τ) was determined to be 2.1 ± 0.2 s. (Inset) zoom-in view of lifetime histogram. The distribution of binding times, which means by stable binding of XPC-RAD23B to CPDs, is shown in the gray histogram. The total number of molecules (N) analyzed for binding times was 109.

To gain insight into how XPC-RAD23B identifies DNA lesions during 1D diffusion, we examined the behavior of XPC-RAD23B on CPD-containing λ-DNA, where three repeats of CPDs were inserted into a specific region in a specially engineered λ-DNA (λ-I3) (Supplementary Figure S1). We tested the binding of XPC-RAD23B to CPD at physiological salt concentration (150 mM NaCl), where the immobile species are suppressed (Figure 2B). In the single-tethered DNA curtain, XPC-RAD23B specifically located the CPD sites (green arrow) on λ-DNA (Figure 5A). The binding distribution histogram displays a single peak around the position of the CPDs on the λ-DNA, demonstrating that XPC-RAD23B preferentially binds to CPDs (Figure 5B). We then examined how XPC-RAD23B identifies CPDs during 1D diffusion. The population of immobile species on CPD dramatically increased while it was still suppressed on non-CPD regions. We therefore considered the immobile species at the CPD sites as CPD-binding state of XPC-RAD23B (Supplementary Figure S8). As shown in kymographs, XPC-RAD23B found CPDs either by 1D movement including diffusive and constrained motion or direct binding (Figure 5C). Interestingly, the population of constrained motion to CPDs was higher than that of diffusive motion to CPDs because our CPD sites located in the AT-track-rich area (Figure 5D). Conversely, the portion of direct binding is just 8.5% (Figure 5D). Because our spatial resolution could not measure the motion only within 1 kbp, we could not exclude the possibility of the short-distance diffusion within one pixel along with direct binding.

In conclusion, our results suggest that XPC-RAD23B identifies DNA lesions via 1D movement on DNA. In many kymographs, XPC-RAD23B did not bind CPDs upon the first encounter and missed the lesions several times (top of Figure 5C). We estimated the CPD recognition efficiency, which was calculated by how many times XPC-RAD23B encountered CPD sites until it bound to CPD (Supplementary Data). For that, we considered only diffusive motion because we could not measure the frequency with which XPC-RAD23B encountered CPDs by direct binding and constrained motion. In Figure 5E, XPC-RAD23B recognized triple CPDs with 25.9% efficiency. Simply approximated, the recognition efficiency for single CPD might be 8.6%, which may be an underestimation. We found that some XPC molecules stably bound to CPDs (top and third of Figure 5C), whereas others diffused away following the initial binding to CPDs (bottom of Figure 5C). Such biphasic kinetics of XPC on CPD became more evident through lifetime analyses. The stably-bound XPC molecules stayed on CPD mostly longer than 50 s (Figure 5F). By contrast, the lifetime of XPC molecules transiently bound to CPDs was about 2.1 ± 0.2 s, which is 25 times shorter than stable binding (Figure 5F).

## DISCUSSION

### The motion of XPC-RAD23B along DNA is heterogenous

Our studies using single-molecule DNA curtains showed that XPC-RAD23B initially binds to random sequences on DNA and then moves on DNA via 1D diffusion. The 1D motion of XPC-RAD23B is divided into diffusive motion and constrained motion that includes an immobile state. Constrained motion has also been observed for yeast ortholog Rad4-Rad23 by Kong *et al.*, and these authors suggested a model that a conformational change of Rad4-Rad23 upon encountering a CPD enabled the protein to surveil the damaged region (±1–2 kbp) for additional lesions (13). Although the model is intriguing, it is unclear what the structural basis for this conformational change would be, considering that the structure of Rad4 on CPD is very similar to that on undamaged DNA (11) and how it would relate to the kinetic gating model proposed based on structural and kinetic studies.

The studies with Rad4 further revealed that a fraction of the protein diffuses in the constrained mode on undamaged DNA and that no transitions between different types of motion were observed. If the damage would specifically induce the constrained motion in addition to immobilization of Rad4, one would expect transitions between different types of motion could be observed. By contrast, we did observe the transitions in the present study and we cannot exclude the possibility that they may be due to differences between the XPC and Rad4 proteins or buffer conditions.

**Figure 6.**
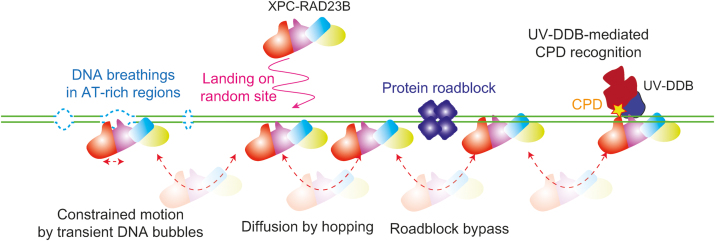
Model for lesion search mechanism of XPC-RAD23B. XPC-RAD23B binds random sequences of DNA and diffuses along DNA via hopping, which facilitates the bypass of protein obstacles on DNA for rapid search for DNA lesions. When XPC-RAD23B encounters AT-rich regions, where transient DNA opening occurs frequently, it may be transiently trapped restricting its motion. XPC detects CPDs with low efficiency, and hence UV-DDB will facilitate CPD recognition by XPC.

Our data however suggest an alternative explanation as we show that the locations of where the constrained motion occurs on λ-DNA are highly correlated with AT-rich positions (Figure 3 and Supplementary Figure S5), in which the duplex DNA can transiently melt (31,32). Biochemical and structural studies have shown that XPC can bind to the DNA mis-pairs (10–11,26,33), strongly suggesting that transiently unpaired bases in a helix derived from DNA breathing can bind and immobilize XPC during diffusion. This is consistent with a kinetic gating mechanism described for Rad4, in which the DNA helix opening time and the residence time of XPC on DNA are both critical for the binding to DNA lesions or mis-pairs (11). The breathing kinetics for a short AT-stretch (∼10 bp) in duplex DNA is 10–100 μs at 100 mM NaCl (31,34). The resident time of XPC-RAD23B on 10 bp estimated from our diffusion coefficient (*D*_diff_: ∼1.5 × 10^6^ bp^2^/s) is about 33 μs at 100 mM NaCl, which is well within the range to the opening time during DNA breathing, supporting the notion that XPC-RAD23B can identify transiently unpaired DNA while it diffuses along DNA. The transiently formed DNA bubbles, which dominantly occur in the regions where many AT-tracks exist, can restrict the diffusive motion by a series of temporary trapping events and cause the constrained motion (Figure 6). Although the transient DNA bubbles predominantly occur in AT-rich regions, they can be also formed in other DNA sequences. However, the probability that transient DNA bubbles form in random sequences is much lower than in AT-rich regions, and hence XPC would be rarely trapped and the constrained motion would be suppressed in random sequences. Likewise, immobile state is induced when XPC is trapped by transiently unpaired base pairs within our spatial resolution. Transitions between diffusive and constrained motion occur when XPC enters into or escapes from multiple-transiently unpaired regions.

DNA breathing and XPC diffusion are both dependent on ionic strength. In Figure 2B, the fraction of diffusive species at 150 mM NaCl doubles compared to that at 40 mM NaCl, whereas immobile species are greatly suppressed at the higher salt concentration. The increase of ionic strength suppresses DNA breathing because it weakens the electrostatic repulsion between negative phosphate backbones of DNA (31). At the higher salt concentration, the diffusion of XPC-RAD23B via hopping is increased and hence the protein is less restricted due to the increased hopping distance and the reduced level of DNA duplex opening (Figure 2B).

### XPC-RAD23B searches for local lesions via hopping

The dramatic increase of the diffusion coefficient with increasing salt concentration strongly suggests that XPC-RAD23B diffuses along DNA via hopping. Although XPC-RAD23B dominantly hops along DNA, we cannot completely exclude the possibility that XPC-RAD23B partially slides along DNA. It has been suggested that hopping should be slower than sliding, based on studies for several proteins that diffuse along DNA (20,35–36). Consistent with the theory, the *D*_diff_’s of XPC-RAD23B up to 100 mM NaCl were smaller than those of proteins showing 1D sliding such as hOgg1, *Escherichia coli* MutL and *lac* repressor (20–22). At 150 mM NaCl, *D*_diff_ of XPC-RAD23B became equivalent to that of the sliding proteins, indicating that XPC can search for DNA lesions as rapidly as other diffusing proteins at physiological salt concentrations.

The 1D diffusion via hopping of XPC-RAD23B gives insight into how XPC targets damaged bases on long genomic DNA in the nucleus. First, the intracellular number of XPC per HeLa cell has been estimated to be in the range of 2.5 × 10^4^ ∼ 8 × 10^4^ (37). Taking into consideration the human genome size (∼3 × 10^9^ bp), one XPC molecule needs to survey approximately 10^5^ bp. Finding local lesions out of 10^5^ bp via 3D collision may well take a longer time than via 1D diffusion. Second, XPC does not need to scrutinize every base to identify DNA lesions because XPC senses a local distortion in duplex DNA due to a lesion (10). Monitoring the DNA backbone via hopping appears not to have adverse effect on sensing local distortions. At last, our collision experiments demonstrated that hopping of XPC facilitates to bypass protein obstacles bound to DNA (Figure 4). The bypass probability of greater than 30% is as large as that of other hopping proteins such as Msh2-Msh3, implying that XPC-RAD23B frequently bypasses protein obstacles (23). Conclusively, 1D diffusion via hopping enables XPC to rapidly search for local lesions on long and crowded genomic DNA.

### XPC inefficiently identifies CPDs and binds them with limited stability

CPDs are the most common DNA lesions generated by UV irradiation and these lesions are repaired by NER at a slow rate. We examined how XPC-RAD23B identifies CPDs during 1D diffusion at physiological salt concentration (Figure 5). Based on our results, the recognition efficiency for single CPD is simply approximated as 8.6%, which implies that diffusive XPC-RAD23B binds a CPD after it misses the lesion 11 or 12 times. It is likely that such inefficient recognition is mainly derived from the limited DNA deformation and destabilization by CPDs (26,38). On the other hand, we cannot exclude the possibility that XPC may bypass lesions via hopping.

We inspected the binding stability of XPC-RAD23B on CPDs. Some molecules stably bound to CPDs for a long time (>50 s) while others only bound transiently (∼ 2 s). According to the structure of Rad4 with a CPD, two β-hairpins are inserted through the CPD site and stabilize the Rad4 binding (10). We suggest that the stable binding of XPC-RAD23B results from the tight configuration through β-hairpin insertion and full base flipping. This stable binding will offer sufficient time to recruit the downstream NER factors such as TFIIH to the lesions. By contrast, the transient binding mode is likely too short for the factors to be assembled by XPC at lesions. A laser temperature-jump study proposed two different modes of XPC: one is a fast searching mode and the other is an interrogating mode by twisting DNA (33). As an intermediated state, the interrogation mode bridges diffusion and stable binding at a lesion. Therefore, we propose that the short binding state is likely to be an intermediate state, in which XPC-RAD23B transiently interacts with CPD prior to the stable binding to lesions. Recent computational studies have shown that the DNA opening and productive interactions are notably absent in the initial binding of Rad4 to lesions that are poor NER substrates (39). It will therefore be of great interest to compare the mobility of XPC-RAD23B on substrates that are repaired with higher efficiency than CPDs by NER.

In cells and in the context of chromatin, the recognition of CPDs by XPC is facilitated by the UV-DDB-containing ubiquitin ligase complex. UV-DDB has the ability to recognize CPDs in the context of chromatin (40–42). The handover of the lesion from UV-DDB to XPC-RAD23B is a complex process that involves ubiquitination of DDB2, XPC and chromatin components and has not yet been fully recapitulated at the biochemical level. While single molecule investigations of this process are therefore not yet achievable, studying the handover from UV-DDB to XPC presents an exciting direction for the future.

## CONCLUSION

Human XPC protein needs to find diverse DNA lesions in genomic DNA that is composed of billions of base pairs and covered with numerous proteins. Our study elucidated how human XPC searches for local DNA lesions, especially CPDs and suggests how this occurs in genomic DNA. As summarized in Figure 6, XPC makes initial contact with random sequences of DNA and diffuses along DNA via hopping, facilitating the scanning of DNA by bypassing protein obstacles. The motion of XPC can sometimes be restricted within AT-rich regions through the interactions with transient bubbles in the DNA duplex. Although XPC detects poor substrates CPDs inefficiently, another factor UV-DDB enhances the CPD binding of XPC.

## Supplementary Material

gkz629_Supplemental_Files
