# Supplementary material for: Single-molecule visualization reveals the damage search mechanism for the human NER protein XPC-RAD23B
Source: Nucleic Acids Res. 2019 Aug 2;47(16):8337–47. doi: 10.1093/nar/gkz629 (PMC6895271; doi:10.1093/nar/gkz629)
Supplement: gkz629_Supplemental_Files [file gkz629_supplemental_files.zip › NAR_SupplementaryData_Revision_NAR-01904-H-2019.docx]

Supplementary Data for:

**Single-molecule visualization reveals the damage search mechanism for the human NER protein XPC-RAD23B**

Na Young Cheon^1^, Hyun Suk Kim^2^, Jung-Eun Yeo^2^, Orlando D. Schärer^1, 2^, and Ja Yil Lee^1,2*^

*^1^School of Life Sciences, Ulsan National Institute of Science and Technology, Ulsan, 44919, Republic of Korea; ^2^Center for Genomic Integrity, Institute of Basic Science, Ulsan, 44919, Republic of Korea*

**To whom correspondence should be addressed:* [*biojayil@unist.ac.kr*](mailto:biojayil@unist.ac.kr)

**Table of contents**

**Supplementary Methods**

1. Purification and fluorescent labeling of proteins
2. *In vitro* NER assay
3. Electrophoretic mobility shift assay (EMSA)
4. Preparation of lesion-containing lambda DNA
5. Single-molecule DNA curtain assay

**Supplementary Figures**

**Figure S1.** Preparation of CPD-containing λ-DNA

**Figure S2.** SDS-PAGE analysis of purified recombinant XPC-RAD23B and activity test of XPC-RAD23B using *in vitro* NER assay and electrophoretic mobility shift assay

**Figure S3.** Different types of motion of XPC-RAD23B at 100 mM and 150 mM NaCl

**Figure S4.** Lateral displacement of XPC-RAD23B on DNA

**Figure S5.** Analyses for the constrained motion and immobile state

**Figure S6.** Analysis for the diffusive and constrained motion

**Figure S7.** Collision between XPC-RAD23B and EcoRI^E111Q^

**Supplementary Table1.** List of oligomers and their sequences

**References**

**Supplementary Movies**

**Movie S1.** [Diffusive motion](file:///E:\UNIST\Papers\천나영\XPC_Rad23B\Manuscript\NAR_version\RevisedVersion\Movie_S1_Diffusive_Motion.mp4)

**Movie S2.** [Immobile species](file:///E:\UNIST\Papers\천나영\XPC_Rad23B\Manuscript\NAR_version\RevisedVersion\Movie_S2_Immobile_Species.mp4)

**Movie S3.** [Constrained motion](file:///E:\UNIST\Papers\천나영\XPC_Rad23B\Manuscript\NAR_version\RevisedVersion\Movie_S3_Constrained_Motion.mp4)

**Supplementary Methods**

**Purification and fluorescent labeling of proteins**

*1. Purification and fluorescent labeling of XPC-RAD23B*

XPC-RAD23B with 3xFLAG at amino-terminus of XPC was expressed in Sf9 insect cells (0.4 L culture). The harvested cells were resuspended in 40 mL of Lysis buffer (10 mM PBS [7.4], 500 mM NaCl, 1 mM PMSF, 0.3% NP-40, PI tablet, and 1 mM βME) and then were lysed by Dounce homogenizer and clarified by ultracentrifugation (40,000 g for 30 min). The clarified lysates were incubated with anti-FLAG M2 agarose bead (Sigma, A2220) for 3.5 hours. The beads were collected by low speed centrifugation and washed twice with 10 ml and four times with 2 ml of washing buffer (10 mM PBS [7.4], 500 mM NaCl, and 0.1% NP-40). Then the beads were stacked in gravity flow column. The XPC-RAD23B was eluted 6 times with one column volume (CV) of 0.2 mg/ml of 3xFLAG peptide (F4799, Sigma) in Lysis buffer. The eluted proteins were subsequently purified through gel filtration (HiLoad16/600 Superdex 200, Pharmacia), which was pre-equilibrated in GF buffer (25 mM potassium phosphate [7.6], 200 mM NaCl, 10% glycerol, and 5 mM βME). The eluted XPC-RAD23B was further purified and concentrated by heparin column (1 mL of HiTrap Heparin, Pharmacia). The proteins were eluted by a linear and ten CVs of the gradient from 0.2 M to 1.5 M of NaCl in GF buffer. The protein eluants were then dialyzed overnight against storage buffer (25 mM potassium phosphate [7.6], 100 mM NaCl, 10% glycerol, and 5 mM βME). The final products were snap-frozen in liquid nitrogen and stored at -80°C until use. Protein concentration was measured by Bradford assay.

For the fluorescence labeling of the purified XPC-RAD23B, FLAG-antibody (F3165, Sigma) was conjugated with the quantum dot (Qdot) using Qdot Antibody Conjugation Kit (S10454, Thermo Fisher Scientific). The purified FLAG-tagged XPC-RAD23B was incubated with FLAG-antibody conjugated Qdots on ice for at least 15 min. To ensure that each Qdot has only one XPC-RAD23B, Qdot and XPC-RAD23B was incubated at 40:1 molar ratio.

*2. Purification and fluorescent labeling of protein obstacles*

For the collision with XPC-RAD23B, a catalytically inactive EcoRI mutant (EcoRI^E111Q^) with 3xFLAG peptides at amino-terminus and intein-tag at carboxyl-terminus was purified as previously described (1). Intein-tagged EcoRI^E111Q^ was transformed to BL21(DE3) *E coli* strain and grown in 2 L of LB media supplemented with carbenicillin. The protein was induced with 1 mM IPTG (isopropyl β-D-1-thiogalactopyranoside) at OD_600_ ~ 0.6 and then further expressed at 16°C for at least 16 hours. Harvested cells were resuspended in 20 mM Tris-HCl [8.5], 500 mM NaCl, 1 mM EDTA, 0.1% Triton X-100, 5% glycerol, 1 mM PMSF (Phenylmethanesulfonyl fluoride), and 1 mM TCEP (Tris(2-carboxyethyl)phosphine hydrochloride) supplemented with protease inhibitor cocktail (78439, Halt, Thermo Fisher Scientific). All the following purification steps were done at 4°C. The cells were lysed by sonication and then spun down by ultracentrifugation at 35,000 g for 30 min. The clarified lysates were loaded into 5 ml of chitin-resin (S6651L, NEB) column through gravity flow. After washing with 20 ml of washing buffer (20 mM Tris-HCl [8.5], 500 mM NaCl, and 1 mM EDTA), the intein was overnight cleaved on the column by the cleavage buffer (20 mM Tris-HCl [8.5], 500 mM NaCl, 50 mM DTT, and 1 mM EDTA). Then EcoRI^E111Q^ was eluted by 40 mM Tris-HCl [7.5], 300 mM NaCl, and 10 mM βME, 0.1 mM EDTA, 50% glycerol, and 0.15% Triton X-100. The eluants were aliquoted and snap-frozen in liquid nitrogen for -80°C storage until use. For the collision experiments, 3xFLAG-tagged EcoRI^E111Q^ and FLAG antibody-conjugated Qdot (605 nm emission) were conjugated at 1:10 molar ratio on ice for at least 15 min.

***In vitro* NER assay**

Cell extracts from XPC-deficient XP4PASV cells and a plasmid containing a 1,3-intrastrand cisplatin adduct were prepared as described (2,3). 3 μl of XP-C cell extract (20 mg/ml) in 100 mM KCl was combined with 2 μl of 5x repair buffer (200 mM Hepes-KOH [7.8], 25 mM MgCl_2_, 110 mM phosphocreatine (di-Tris salt, Sigma), 10 mM ATP, 2.5 mM DTT and 2 mg/ml BSA), 0.2 μl of 2.5 mg/ml creatine phosphokinase (rabbit muscle CPK, Sigma), and either purified XPC and/or NaCl (to a final NaCl concentration of 70 mM). The mixture was incubated at 30°C for 10 min. Then 1 μl of closed circular plasmid DNA (50 ng) containing a 1,3-intrastrand cisplatin crosslink was added to the mixture, which was further incubated at 30°C for 45 min. After placing the samples on ice, 0.5 μl of 1 μM 35-mer oligonucleotide that anneals to the excision product (5’-GGG GGA AGA GTG CAC AGA AGA AGA CCT GGT CGA CCp-3’) was added to the mixture, which was then heated to 95°C for 5 min. The samples were allowed to cool down to 23°C for 15 min to allow for annealing of the oligonucleotide to the substrate. 1 μl of a Sequenase [⍺-^32^P]-dCTP mix (0.5 units of Sequenase and 2.5 μCi of [⍺-^32^P]-dCTP per reaction) was added. After incubating at 37°C for 3 min, 1.2 μl of dNTP mix (100 μM of each dATP, dTTP, dGTP; 50 μM dCTP) was added and the mixture incubated for another 12 min to extend the excised product with radiolabeled dCTP. The reactions were stopped by adding 8 μl of loading dye (90% formamide and 10 mM EDTA) and heating at 95°C for 5 min. The samples were loaded on a 14% sequencing gel (0.5x TBE) and run at 45 W for 2.5 hrs. The reaction products were visualized using a PhosphorImager (Typhoon RGB, GE Healthcare).

**Electrophoretic mobility shift assay (EMSA)**

To test the activity of XPC-RAD23B, we performed electrophoretic mobility shift assay (EMSA). 39-mer oligomer (λ-I3_WT) and its complementary oligomer labeled with Cy3 (λ-I3_comp_Cy3) were synthesized from Bioneer (South Korea) (Supplementary Table 1). The oligomer containing a CPD (λ-I3_CPD) was synthesized from Gene Link (USA) (Supplementary Table 1). λ-I3_WT or λ-I3_CPD was mixed with λ-I3_comp_Cy3 at 1.2:1 molar ratio. The mixture was heated up to 95°C and slowly cooled down to 23°C for annealing. 10 nM of each annealed DNA was incubated with XPC-RAD23B at different concentrations in 25 mM Tris-HCl [7.5] and 150 mM NaCl at 23°C for 20 min in a dark room to prevent the photobleaching of Cy3. The reactants were analyzed in 5% nondenaturing polyacrylamide gel, which was run at 100 V using 0.5x TBE buffer at 4°C. The gel was then scanned by Typhoon RGB (GE Healthcare). The band intensity was quantified by Gel Analysis tools in ImageJ (NIH). The bound fraction was calculated by dividing the intensity of bound band by the sum of bound and unbound band intensities.

**Preparation of lesion-containing lambda DNA**

*1. λ-I3 preparation*

CPDs were inserted into λ-DNA by following the protocols in the previous literatures (4,5). To this end, the specially engineered λ-I3 was used, which contained three repeated sequences including seven nickase (Nt.Bsp.QI) sites and three NcoI cleavage sites (Figure S1). The λ-I3 was packaged into λ-phage particles using MaxPlax λ extracts (MP5105, Epicentre) and then was infected into *E coli* LE392MP strain. The infected *E coli* cells were spread on LB plate and incubated overnight at 37°C to make plaques. Next day, a chunk of single plaque was taken and mixed with overnight cultured LE392MP cells. The mixture was further grown up in 200 ml of NZCYM broth at 37°C with an agitation at 125 rpm. OD_600_ rose up greater than 1.0 and then suddenly began to drop down because λ-phage particles also grew as cells grew up. When OD_600_ dropped down to about 0.3, 5 ml of chloroform was added to the cell culture, which was further incubated for 15 min. NaCl up to 1 M was added to the cell culture, which was then overnight incubated with 10% PEG 20000 (81300, Sigma) at 4°C with slow tumbling for precipitating λ-phage particles. The λ-phage particles were harvested and resuspended in SM buffer (10 mM Tris-HCl [8.3], 100 mM NaCl, and 10 mM MgCl_2_). RNase A (R4875, Sigma) and DNase I (D5319, Sigma) were treated to remove genomic DNA and RNAs. After treated with 0.05 mg/ml of proteinase K and 1.25% SDS, λ-I3 DNA was precipitated with IPA.

*2. Insertion of lesion-containing oligomers into λ-I3*

To insert CPD-containing oligomer, 3 nM of λ-I3 was treated with 0.6 unit/ul of Nt.Bsp.QI nickase (New England Biolabs) at 65°C to induce a gap at a specific location on the λ-I3. Nickases were degraded by 0.1 mg/ml of proteinase K, which was then heat-inactivated at 65°C. 500-fold excessive oligomers containing a CPD (λ-I3_CPD), Lambda biotin-R (or Lambda biotin-L) and Lambda dig-L (or Lambda dig-R) were simultaneously ligated with the gapped λ-I3. After heat inactivation, the λ-DNA was purified with S-400 spin column (Illusta MicroSpin^TM^ S-400, GE Healthcare). The insertion was confirmed by NcoI digestion. The λ-DNA without CPDs was cleaved by NcoI, whereas the CPD-containing λ-DNA was not (Figure S1C).

**Single-molecule DNA curtain assay**

*1. Total internal reflection fluorescence microscope (TIRFM)*

Prism-type total internal reflection fluorescence microscope (TIRFM) was custom-built with Nikon Eclipse Ti-2. The solid-state 488-nm (200 mW, OBIS, Coherent Laser) was used to excite Qdots. The laser beam passed through a dove prism and was totally reflected at the boundary between a fused-silica slide and buffer solution with an incident angle beyond the critical angle, generating an evanescent field. The fluorescence from Qdots was collected by a 60x water-immersion objective lens (CFI Plan Apo VC 60XWI, Nikon). The fluorescence was then split by dichroic mirror (ZT561rdc-xr or T635lpxr-UF1, Chroma Technology) and imaged on each of two EM-CCD cameras (iXon 897, Andor Technology). Emission filters (ET500lp for YOYO-1, ET590.50m for 605 nm Qdot, and ET650lp for 705 nm Qdots, Chroma Technology) were used to block the excitation laser light. The data were collected by the NIS-Element software (Nikon).

*2. Flowcell preparation for the DNA curtain assay*

The flowcell for DNA curtain assay was prepared by following the previous protocol (1,6). Fused-silica slides with two holes were cleaned by successive treatment with 2% Hellmanex III (Sigma), acetone, 1 M sodium hydroxide, and DI water. The slides were dried with N_2_ gas for patterning nano-barriers. The narrow and wide (pentagonal) chromium nano-barriers were fabricated on each cleaned slide through the following nano-patterning process: the spin-coating of e-beam resistor (PMMA), electron beam lithography, development, chromium deposition by electron beam evaporator, and lift-off by acetone. Then the flowcell with a microchannel was constructed by gluing the nano-barrier slide and a coverslip with a double-sided tape. Nanoports (IDEX) were attached on the holes to connect the flowcell with a fluidic system. The flowcell was rinsed with DI and lipid buffer (20 mM Tris-HCl [8.0] and 100 mM NaCl). Liposomes consisting of DOPC (1,2-dioleoyl-*sn*-glycero-phosphocholine), 0.5% biotinylated-DPPE (1,2-dipalmitoyl-*sn*-glycero-3-phosphoethanolamine-N-(cap biotinyl)), and 8% mPEG 2000-DOPE (1,2-dioleoyl-*sn*-glycero-3-phosphoethanolamine-N-[methoxy(polyethylene glycol)-2000]) (Avanti-Polar Lipids) were deposited on the microchannel surface. The lipid bilayer was promoted on the surface by 20 min incubation after free liposomes were washed with lipid buffer. 0.1 mg/ml of anti-digoxigenin (11214667001, Roche) in lipid buffer was added to the flowcell and nonspecifically adsorbed on the wide chromium nano-barrier. BSA buffer (40 mM Tris-HCl [8.0], 50 mM NaCl, 2 mM MgCl_2_, and 0.4% BSA) was injected to further passivate the surface that was not covered with lipid bilayer. 1 mg/ml streptavidin in BSA buffer was subsequently introduced into the chamber. λ-DNA with biotin and digoxigenin at either end was anchored on biotinylated lipid via biotin-streptavidin linkage. The flowcell was connected to a fluidic system consisting of a syringe pump and a 6-way injection valve and then placed on the TIRFM. Under the continuous buffer flow (0.3 ml/min), DNA molecules were moving on lipid bilayer along the flow and then stretched with the biotinylated end stuck at the narrow nano-barrier. The other end tagged with digoxigenin was tethered to the wide nano-barrier coated with anti-digoxigenin. Finally, the DNA molecules were doubly-tethered between two barriers and held stretched even in the absence of flow (Figure 1A).

For the single-tethered DNA curtain assay, we used the slide that had only narrow barrier and omitted the anti-digoxigenin step, so that only biotinylated end of λ-DNA was anchored on lipid bilayer. Therefore, to extend λ-DNA molecules, buffer was continuously flowed in.

*3. Imaging of XPC-RAD23B*

XPC-RAD23B tagged with Q-dot was diluted in the XPC buffer (25 mM Tris-HCl [7.5] with 40 mM, 100 mM or 150 mM NaCl) at 23°C. When YOYO-1 was used to stain DNA, 1.6% glucose and 0.1x gloxy were added. 0.5 ~ 1 nM of XPC-RAD23B was injected into DNA curtains. To obtain the initial binding positions, the injection was stopped when a maximum amount of proteins reached the DNA curtains, and then data were collected with 10 Hz of frame rate for 5 min.

*4. Collision between XPC-RAD23B and EcoRI^E111Q^*

3 nM EcoRI^E111Q^ tagged with 605 nm Qdot was injected into the flowcell and incubated with DNA in EcoRI buffer (40 mM Tris-HCl [7.5], 50 mM NaCl, and 2 mM MgCl_2_). After incubation, unbound EcoRI^E111Q^ was completely washed out by XPC buffer with 150 mM NaCl. Then, 1 nM XPC-RAD23B tagged with 705 nm Qdot was injected in the XPC buffer with 150 mM NaCl. Immediately after the maximum amount of XPC-RAD23B arrived at the DNA curtains, the flow was turned off, and images were taken through NIS-elements (Nikon) with 100 msec exposure time for 5 min. The 488-nm laser was used to excite both Qdots because Qdots have a broad range of absorption spectra.

*5. Data analyses for DNA curtain experiments*

All images collected by NIS-Element (Nikon) were converted into 8-bit TIFF format. All analyses were performed by ImageJ (NIH). The particle tracking was carried out by the ImageJ plug-in, MOSAICSuite particle tracker. For the initial binding positions, we chose molecules that began to bind DNA after data acquisition. The initial binding positions were obtained by taking the position coordinates of the first frame for each particle tracking data. The locations of constrained motions were gained from the mean of the restricted fluctuation.

*6.Calculation of diffusion coefficient*

Traces of XPC-RAD23B were obtained by MOSAICSuite particle tracker, which is an Image J plug-in (NIH). Using Matlab (Mathworks), 1D diffusion coefficient (*D*) of each XPC-RAD23B molecule was calculated from mean square displacement (*MSD*), which is mathematically given as

$MSD \left( n, N \right)= \sum_{i=1}^{N-n} \frac{{(Y_{i+n}-Y_{i})}^{2}}{N-n}=2Dn\Delta t$,

where *N* is the total number of frames, *n* is the measurement window ranging from 1 to *N-1*, *Δt* is the time interval between frames, and *Y* is the position of XPC-RAD23B along DNA. The standard deviation (*SD*) in *MSD*, which was used as error, was given as

$SD=\sqrt{\frac{\left( 2Di\Delta t \right)^{2}(2t^{2}+1)}{3i(N-i+1)}}$.

The diffusion coefficient *(D)* was obtained from the linear fitting of *MSD* with the first three data points because the error of *MSD* becomes large as the frame increases. We tested different number of data points for the linear fitting to estimate diffusion coefficients, but there was no significant difference (Figure S6I).

*7.Lifetime analysis*

For the quantitative analysis for the binding kinetics of XPC-RAD23B on CPDs, lifetime analysis was performed. Only for the molecules showing both binding and dissociation at CPDs, the lifetimes were collected from the kymographs. Then the lifetime histogram was built with 1 sec bin size, which was fitted by a single exponential decay function. On the other hand, for the other molecules on CPDs that were already bound from data acquisition or did not dissociate until the end of data collection, the histogram for time staying (binding time) on CPDs was made with 10 sec bin size.

*8. Calculation of CPD recognition efficiency*

To estimate the CPD recognition efficiency, we chose XPC-RAD23B molecules showing the binding to CPDs through diffusive motion. XPC-RAD23B bypasses or reverses when it encounters CPDs. From kymographs, we obtained *N_missing_*, which means by how many times XPC-RAD23B bypasses or reverses before it binds to CPDs. Then XPC finally recognizes CPDs at (*N_missing_* + 1)^th^ attempt. Therefore, the CPD recognition efficiency was calculated from $\frac{1}{(N_{missing}+1)}$.

**Supplementary Figures**

**Figure S1. Preparation of CPD-containing λ-DNA**


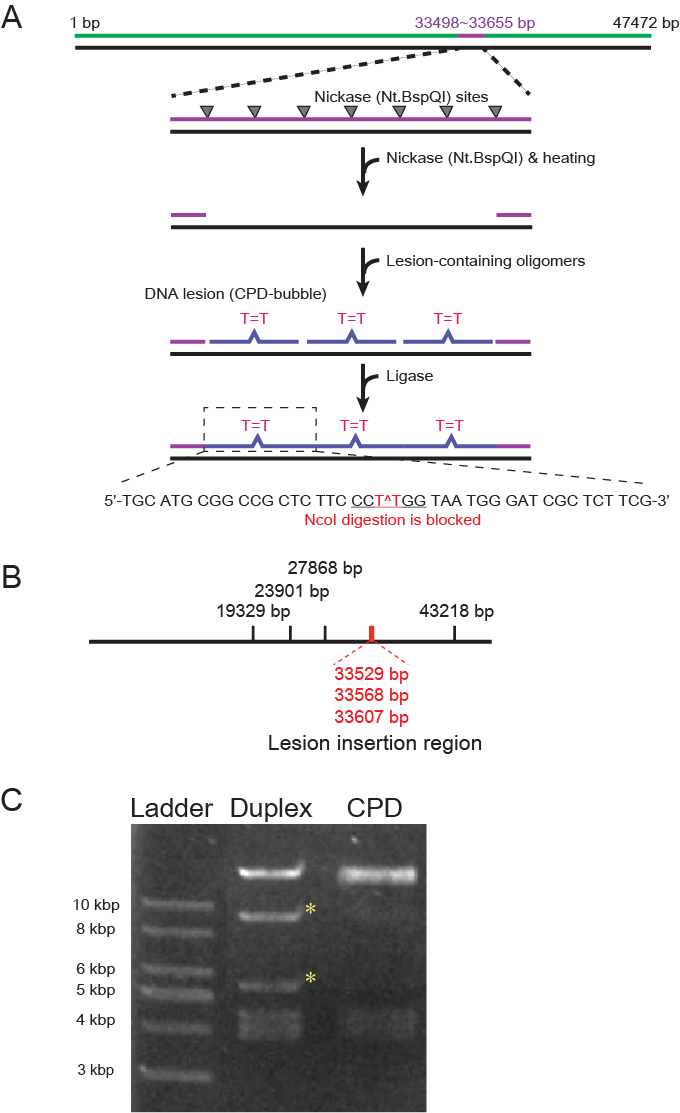


(A) Schematic diagram for strategy to make CPD-containing λ-DNA. Three CPDs were inserted into λ-I3.

(B) Map of NcoI sites in λ-I3, which contains seven NcoI cleavage sites. If lesions are inserted, only four NcoI sites are available for cleavage.

(C) Agarose gel image after NcoI digestion. When CPDs were inserted, there were no fragments of ~5,660 bp and ~9,611 bp (yellow asterisks) because there was no cleavage in the region between 33,498 bp and 33,655 bp.

**Figure S2. SDS-PAGE analysis of purified recombinant XPC-RAD23B and activity test of XPC-RAD23B using *in vitro* NER assay and electrophoretic mobility shift assay**


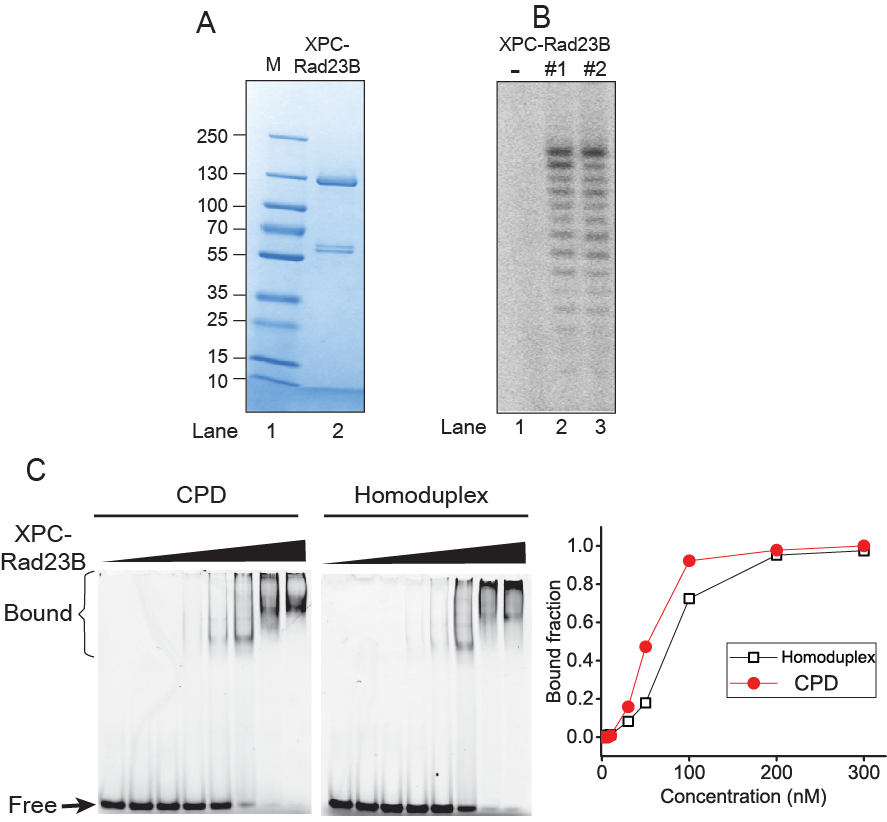


(A) 3xFLAG-XPC-RAD23B complexes were co-expressed and purified from Sf9 insect cells. SDS-PAGE analysis of purified XPC-RAD23B shows the presence of XPC (~135 kDa) and RAD23B (~60 kDa). The gel was stained with Coomassie blue.

(B) XPC-deficient cell extracts were incubated with a plasmid containing a 1,3-intrastrand cisplatin adduct in the absence (lane 1) or presence of wild-type XPCs (batch #1 in lane 2 and batch #2 in lane 3). The reactants were visualized by a fill-in reaction after annealing the excision product to a complementary oligonucleotide with a 4-nt overhang (2). Two different batches of purified XPC-RAD23B complexes were tested (lane 2 and 3).

(C) 5% nondenaturing PAGE was performed with 10 nM of CPD-containing DNA (left) and the undamaged duplex DNA (middle) labeled with Cy5 with increasing XPC-RAD23B concentrations. The relative bound fraction was quantified by Image J (right). The red-filled circles and blank squares represent the CPD-containing DNA and homoduplex DNA, respectively.

**Figure S3. Different types of Motion of XPC-RAD23B at 100 mM and 150 mM NaCl**


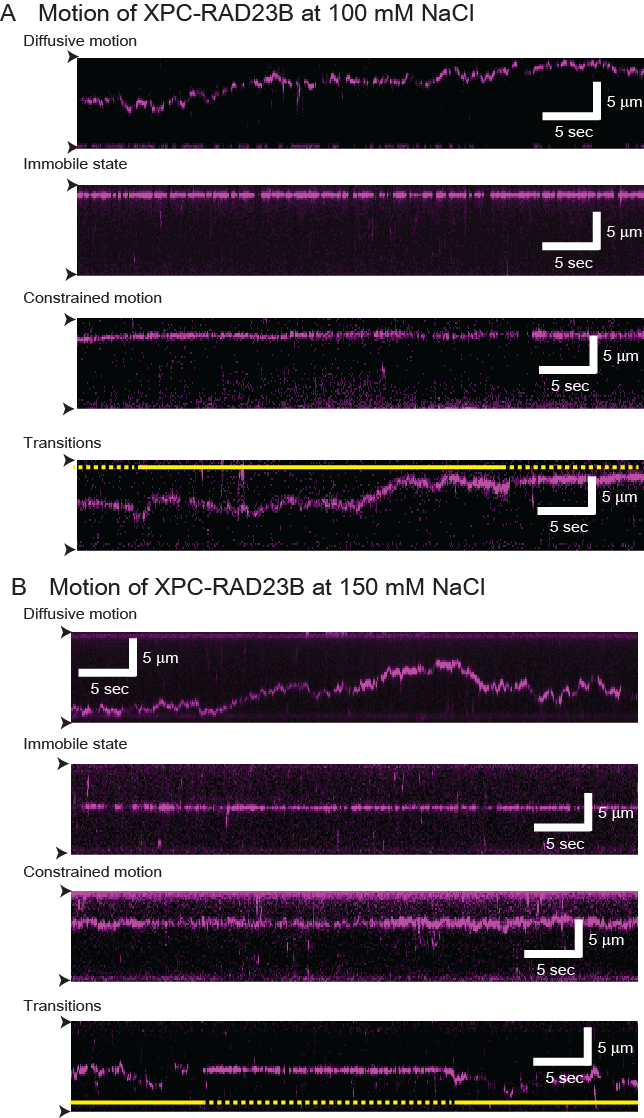


(A) Kymographs for different types of motion of XPC-RAD23B at 100 mM NaCl. From top to bottom, diffusive motion, immobile state, constrained motion, and transition between two different states are displayed. (B) Kymographs for different types of motion of XPC-RAD23B at 150 mM NaCl. From top to bottom, diffusive motion, immobile state, constrained motion, and transition between two different states are displayed. The yellow solid and dashed lines represent diffusive motion and constrained motion, respectively.

**Figure S4. Lateral displacement of XPC-RAD23B on DNA**


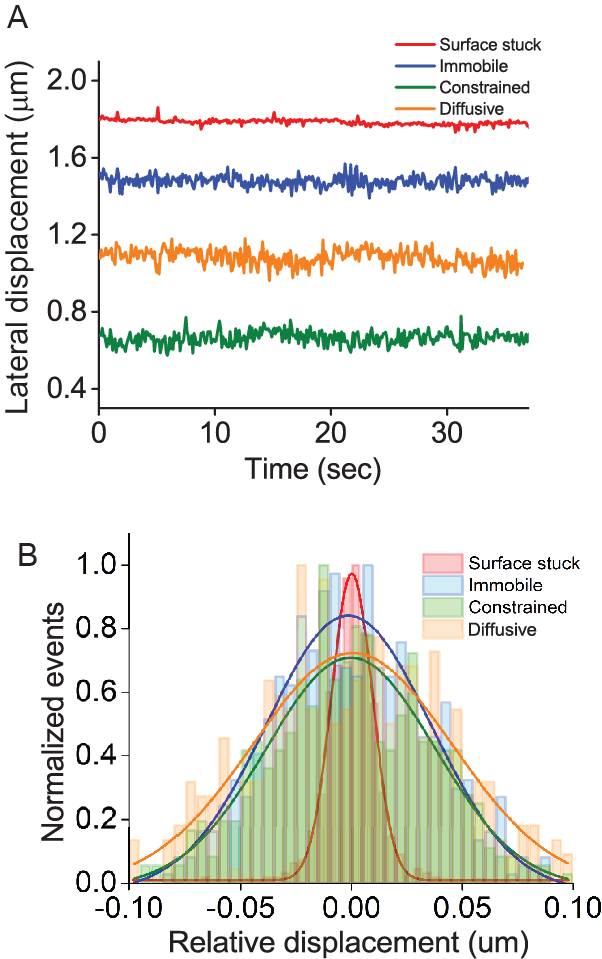


(A) Time traces of diffusive (dark green), constrained (orange), immobile (blue), and surface-stuck (red) molecules. The traces were extracted by the single-molecule particle tracking.

(B) Histograms of relative displacements of adjacent time fracmes for lateral movement of diffusive (dark green), constrained (orange), immobile (blue), and surface-stuck (red) molecules. Each histogram was fitted by a single Gaussian function (solid lines). The center position of all fitted Gaussian functions is zero. The fitted width of each Gaussian function is 0.09 ± 0.01 μm, 0.11 ± 0.02 μm, 0.09 ± 0.01 μm, 0.02 ± 0.00 μm for diffusive, constrained, immobile, and surface-stuck molecules, respectively.

**Figure S5. Analyses for the constrained motion and immobile state**


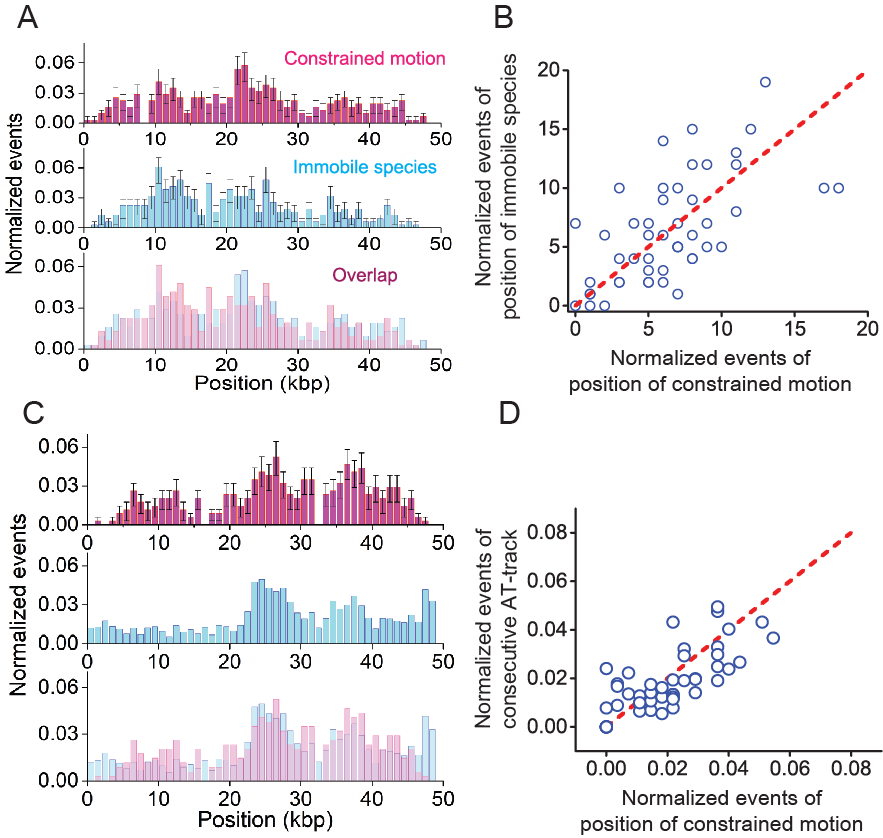


(A) Histogram for positions of constrained motion and immobile species. Top: histogram for the positions of constrained motion, middle: histogram for the positions of immobile species, and bottom: the overlap of the above two histograms.

(B) The correlation analysis of positions of constrained motion and immobile species. The Pearson’s correlation coefficient is 0.6 with 10^-7^ p-value. The red dashed line represents the perfect positive correlation.

(C) Histogram for positions of constrained motion and consecutive AT-tracks (> 4 bp) in the reversed λ-DNA. Top: histogram for the positions of constrained motion, middle: histogram for consecutive AT-tracks (bin size: 1 kbp), and bottom: the overlap of the above two histograms.

(D) Correlation analysis of the positions of constrained motion and the locations of consecutive AT-tracks in the reversed λ-DNA. Pearson correlation coefficient is 0.7 with 10^-8^ p-value. The red dashed line represents the perfect positive correlation.

**Figure S6. Analysis for the diffusive and constrained motion**


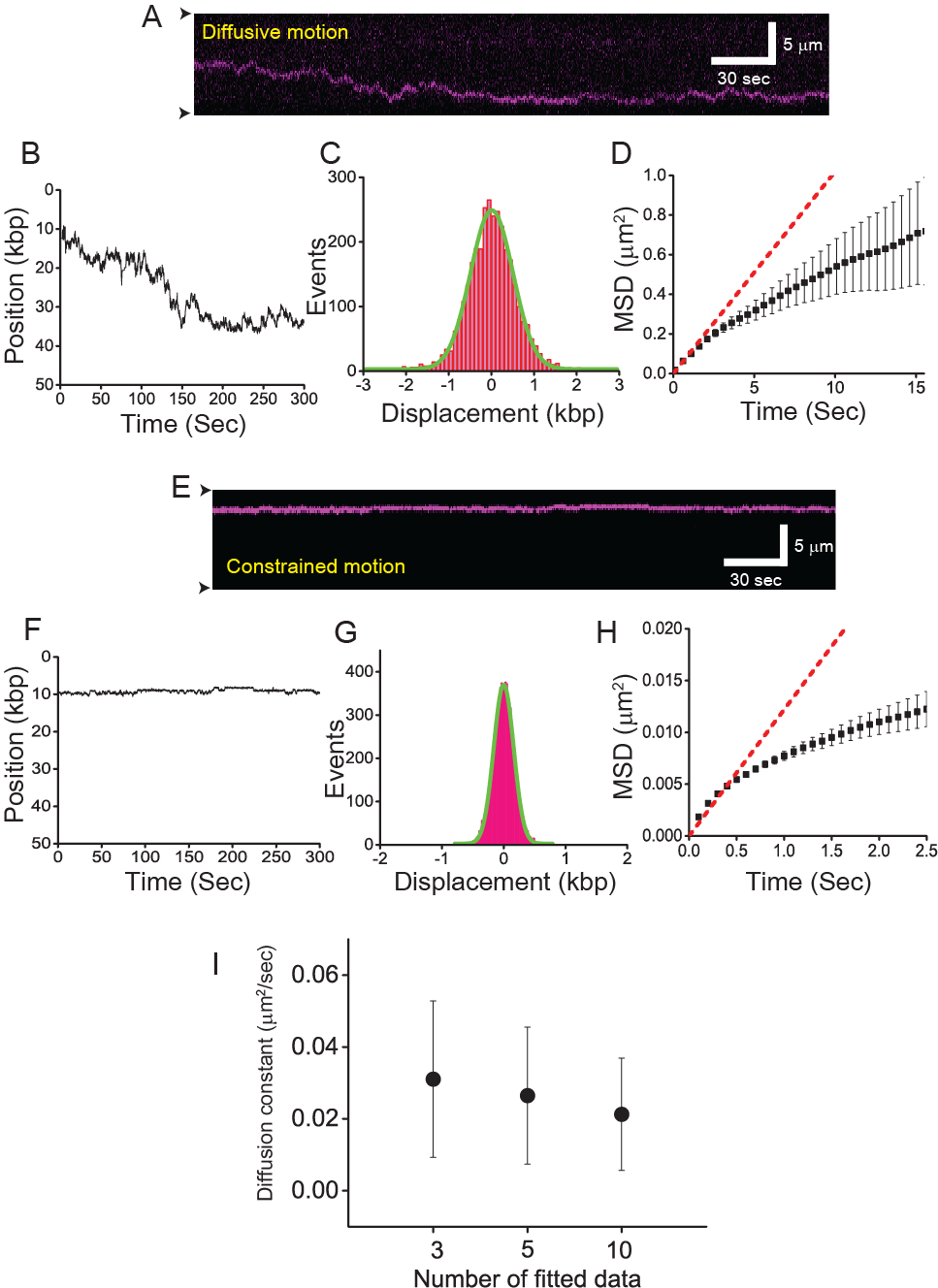


(A) Representative kymograph for diffusive motion (adopted from top of Figure 1C).

(B) Time trace that was obtained from the particle tracking for the diffusive motion.

(C) Histogram for the relative displacements of adjacent time frames, which was extracted from the time trace in (B). The histogram was fitted by a single Gaussian function, the center of which was placed around zero.

(D) MSD for the diffusive motion was calculated as described in Supplementary Data. The diffusion coefficient (*D_diff_*) was estimated from the linear fitting (red dotted line) of the first three points.

(E) Representative kymograph of constrained motion (adopted from the 3^rd^ top of Figure 1C).

(F) Time trace that was obtained from the particle tracking for the constrained motion.

(G) Histogram for the relative displacements of adjacent time frames, which was extracted from the time trace in (F). The histogram was fitted by a single Gaussian function, the center of which was placed around zero.

(H) MSD for the constrained motion. The diffusion coefficient (*D_cons_*) was estimated from the linear fitting (red dotted line) of the first three data points.

(I) Diffusion coefficients according to the number of data points used for the linear fit in MSD. As the number of data points for fitting increased, the diffusion coefficients barely change.

**Figure S7. Collision between XPC-RAD23B and EcoRI^E111Q^**


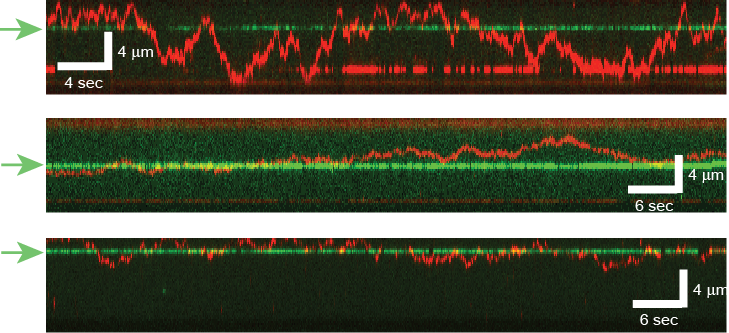


Kymographs displaying the collision events between single XPC-RAD23B (red) and EcoRI^E111Q^ (green) placed on its cognate site. The green arrows represent the cognate sites of EcoRI in λ-DNA.

**Figure S8. Relative fraction of different types of motion on CPD-containing λ-DNA**


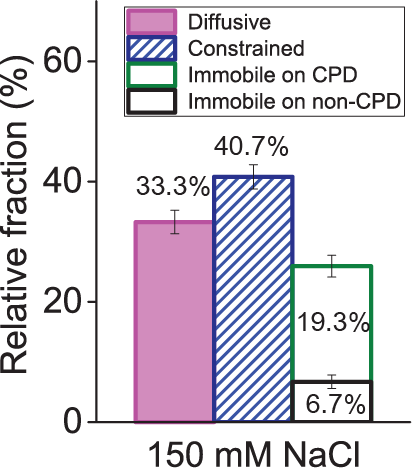


The error bars were obtained by the standard deviation of multinomial distribution.

**Supplementary Table 1. List of oligomers and their sequences**

| Name | Sequence |
| --- | --- |
| Lambda biotin-L | 5’-Phos-GGG CGG CGA CCT-biotin-3’ |
| Lambda biotin-R | 5’-Phos- AGG TCG CCG CCC-biotin-3’ |
| Lambda dig-L | 5’-Phos-GGG CGG CGA CCT-digoxigenin-3’ |
| Lambda dig-R | 5’-Phos- AGG TCG CCG CCC-digoxigenin-3’ |
| λ-I3_WT† | 5’-Phos-TGC ATG CGG CCG CTC TTC CCA TGG TGC GAT CGC TCT TCG -3’ |
| λ-I3_CPD * | 5’-Phos-TGC ATG CGG CCG CTC TTC CC**T^T**GG TGC GAT CGC TCT TCG -3’ |
| λ-I3_comp_Cy3 | 5’-Cy3-CGA AGA GCG ATC GCA CCA TGG GAA GAG CGG CCG CAT GCA-3’ |

† The sequence in green (CCA TGG) represents the NcoI cleavage site, which will be protected by the insertion of CPDs.

* The bold **T^T** represents CPD.

**References**

1. Lee, J.Y., Finkelstein, I.J., Arciszewska, L.K., Sherratt, D.J. and Greene, E.C. (2014) Single-Molecule Imaging of FtsK Translocation Reveals Mechanistic Features of Protein-Protein Collisions on DNA. *Mol Cell*, **54**, 832-843.

2. Mahmud K. K. Shivji, J.G.M., Isao Kuraoka, and Richard D. Wood. (1999) Dual-incision assays for nucleotide excision repair using DNA with a lesion at a specific site. *Methods in Molecular Biology*, **113**, 313-392.

3. Dunand-Sauthier, I., Hohl, M., Thorel, F., Jaquier-Gubler, P., Clarkson, S.G. and Scharer, O.D. (2005) The spacer region of XPG mediates recruitment to nucleotide excision repair complexes and determines substrate specificity. *J Biol Chem*, **280**, 7030-7037.

4. Gorman, J., Wang, F., Redding, S., Plys, A.J., Fazio, T., Wind, S., Alani, E.E. and Greene, E.C. (2012) Single-molecule imaging reveals target-search mechanisms during DNA mismatch repair. *P Natl Acad Sci USA*, **109**, E3074-E3083.

5. Kim, Y., de la Torre, A., Leal, A.A. and Finkelstein, I.J. (2017) Efficient modification of lambda-DNA substrates for single-molecule studies. *Sci Rep-Uk*, **7**.

6. Lee, J.Y., Finkelstein, I.J., Crozat, E., Sherratt, D.J. and Greene, E.C. (2012) Single-molecule imaging of DNA curtains reveals mechanisms of KOPS sequence targeting by the DNA translocase FtsK. *P Natl Acad Sci USA*, **109**, 6531-6536.
